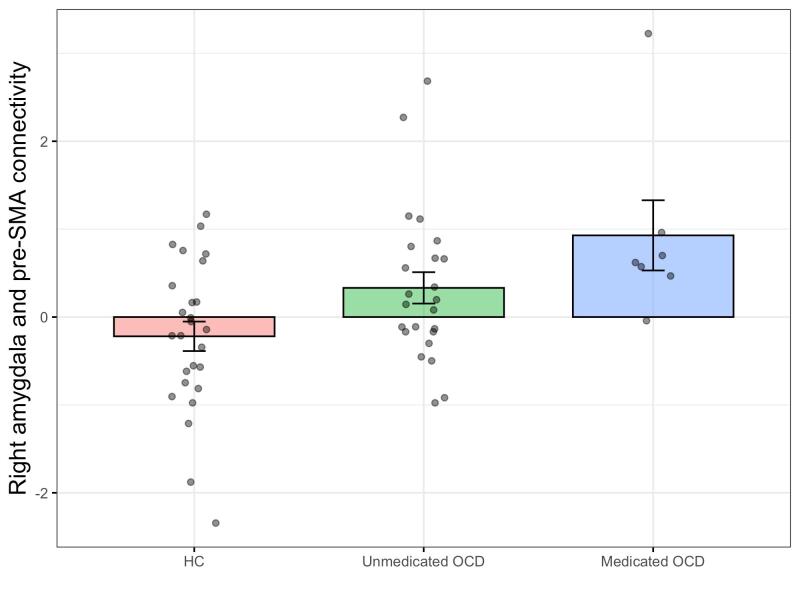# Stable inhibition-related inferior frontal hypoactivation and fronto-limbic hyperconnectivity in obsessive–compulsive disorder after concentrated exposure therapy

**DOI:** 10.1016/j.nicl.2020.102460

**Published:** 2020-10-13

**Authors:** Anders Lillevik Thorsen, Stella J. de Wit, Pernille Hagland, Olga Therese Ousdal, Bjarne Hansen, Kristen Hagen, Gerd Kvale, Odile A. van den Heuvel

**Affiliations:** a Bergen Center for Brain Plasticity, Haukeland University Hospital, Bergen, Norway; b Department of Clinical Psychology, University of Bergen, Bergen, Norway; c Department of Radiology, Haukeland University Hospital, Bergen, Norway; d Center for Crisis Psychology, University of Bergen, Bergen, Norway; e Psychiatric Department, Hospital of Molde, Molde, Norway; f Amsterdam UMC, Vrije Universiteit Amsterdam, Department of Psychiatry, Department of Anatomy and Neurosciences, Amsterdam Neuroscience, Amsterdam, Netherlands

**Keywords:** OCD, Response inhibition, Connectivity, Inferior frontal gyrus, Hypoactivation, Treatment

## Abstract

Response inhibition has previously been suggested as an endophenotype for obsessive–compulsive disorder (OCD), evidenced by studies showing worse task performance, and altered task-related activation and connectivity. However, it’s unclear if these measures change following treatment. In this study, 31 OCD patients and 28 healthy controls performed a stop signal task during 3 T functional magnetic resonance imaging before treatment, while 24 OCD patients and 17 healthy controls were rescanned one week and three months after concentrated exposure and response prevention over four consecutive days using Bergen 4-Day Format. To study changes over time we performed a longitudinal analysis on stop signal reaction time and task-related activation and amygdala connectivity during successful and failed inhibition. Results showed that there was no group difference in task performance. Before treatment, OCD patients compared to controls showed less inhibition-related activation in the right inferior frontal gyrus, and increased functional connectivity between the right amygdala and the right inferior frontal gyrus and pre-supplementary motor area. During error-processing, OCD patients versus controls showed less activation in the pre-SMA before treatment. These group differences did not change after treatment. Pre-treatment task performance, brain activation, and connectivity were unrelated to the degree of symptom improvement after treatment. In conclusion, inferior frontal gyrus hypoactivation and increased fronto-limbic connectivity are likely trait markers of OCD that remain after effective exposure therapy.

## Highlights

• Less IFG activation and more fronto-limbic connectivity was found in OCD.
• IFG hypoactivation and fronto-limbic hyperconnectivity persisted after exposure therapy.
• Activation and connectivity before treatment did not predict treatment outcome.

## 1 Introduction

Obsessive-compulsive disorder (OCD) is characterized by intrusive obsessions and repetitive compulsions (American Psychiatric Association, 2013). The disorder affects 1–3% of the population, is related to substantial impairment in personal, family and work life, and often remains chronic if untreated (Stein et al., 2019).

OCD patients have difficulty stopping rituals or ruminating once they have started, and tasks measuring the ability to cancel behaviors may be relevant to study the neurobiological processes underlying these symptoms (van Velzen et al., 2014). Response inhibition involves suppressing and cancelling actions in order to efficiently complete a task. Different paradigms exist that assess the subprocesses of inhibition, ranging from interference control tasks (e.g. Flanker task), action withholding tasks (e.g. Go/No-go task), and action cancellation tasks (e.g. Stop signal task; SST) (van Velzen et al., 2014). Meta-analyses have shown that OCD patients show small to moderate difficulties in response inhibition relative to healthy controls, with the largest difference in action cancellation (Snyder et al., 2015).

Functional neuroimaging studies have related OCD to subtle alterations in cortico-striato-thalamo-cortical, fronto-parietal, and fronto-limbic circuits (Stein et al., 2019). A recent meta-analysis (Norman et al., 2019) of response inhibition found that OCD patients compared to controls showed less inhibition-related activation in areas of the fronto-parietal and ventral attention networks, including the dorsal anterior cingulate cortex (dACC) and anterior insula. Hyperactivation was found in the premotor, orbitofrontal, parietal, and temporal cortices, as well as the thalamus and caudate nucleus. OCD patients also showed more error-related activation in cingulo-opercular regions, also including the dACC and pre-supplementary motor area (pre-SMA) (Norman et al., 2019).

In an endophenotype functional magnetic resonance imaging (fMRI) study of the SST in 41 OCD unmedicated patients, 17 unaffected siblings and 37 healthy controls, patients (compared to controls) showed less activation in the right IFG and inferior parietal during inhibition, while both patients and siblings (compared to controls) showed more pre-SMA activation. Pre-SMA activation was suggested to be compensatory since it was related to better inhibitory task performance (shorter stop-signal reaction time; SSRT) (de Wit et al., 2012). Patients and unaffected siblings also showed less connectivity between the left IFG and bilateral amygdala during successful inhibition, which was negatively related to pre-SMA activation (van Velzen et al., 2015). This suggests that both OCD patients and siblings show altered recruitment of inferior frontal and premotor cortices during action cancellation, as well as altered limbic connectivity.

Recommended first-line treatments for OCD include exposure and response prevention (ERP) and serotonin reuptake inhibitors (SRI) (Stein et al., 2019). ERP can be effectively delivered intensively, weekly, individually or in group-settings (Öst et al., 2015). Rapid and sustained recovery after concentrated treatment may be provide a basis for investigating both short- and long-term changes in task performance and inhibition-related brain network function.

There are few studies on how response inhibition and its neural correlates change after successful treatment (Thorsen et al., 2015). Previous findings have been inconsistent, with both increased or unchanged task-related activation (or event related potentials when using EEG) after treatment, and inconsistencies in where the changes were observed (Nabeyama et al., 2008, Nakao et al., 2005, Riesel et al., 2015). It’s therefore unclear if hyperactivation or hypoactivation are trait markers or endophenotypes of OCD, or if they are dependent on the severity of the disorder (de Wit et al., 2012). If these are traits, one would expect that these abnormalities remain after successful treatment. If they are state-related, hyperactivations might increase so that patients resemble unaffected siblings (de Wit et al., 2012), or decrease if compensation is no longer needed. There is also evidence that increased fronto-limbic connectivity may interfere with cognitive control in OCD and unaffected siblings (de Vries et al., 2014, van Velzen et al., 2015). We recently found reduced (normalized) connectivity between the fronto-parietal and limbic networks after treatment using resting-state fMRI (Thorsen et al., 2020), which may suggest that limbic and task-related areas become more independent after treatment.

We here investigated if concentrated ERP leads to changes in performance, task-related brain activation and connectivity during the response inhibition and error processing using the SST. Following a preregistered analysis plan (https://osf.io/ye7q3), we first assessed 31 patients and 28 controls the day before treatment, and assessed changes in 24 patients and 17 healthy controls after one week and after three months. During successful inhibition, we expected to find hypoactivation in the IFG, dACC and parietal cortex, hyperactivation of the pre-SMA, and increased fronto-limbic connectivity in OCD patients before treatment. We also expected more dACC and pre-SMA activation during error processing. After treatment, we expected increased inhibition-related activation in the pre-SMA, IFG, inferior parietal cortex, and dACC, and the fronto-limbic connectivity to normalize. We did not expect error-related activation to decrease after treatment.

## 2 Methods

### 2.1 Participants

The study recruited 35 OCD patients and 31 healthy controls before treatment (See Table 1 for demographics, comorbidity, and medication). Patients were recruited from a specialized outpatient OCD clinic at Haukeland University Hospital, Bergen, Norway, while controls were recruited using bulletin boards, social media, and emails to local businesses. Patients were 18 years or older, had a primary diagnosis of OCD, had a minimum Yale Brown Obsessive Compulsive Scale (Y-BOCS) (Goodman et al., 1989) score of 16, and were fluent in Norwegian. Exclusion criteria for patients were symptoms primarily associated with hoarding, ongoing substance abuse, bipolar disorder or psychosis, suicidal ideation, intellectual disability, or being unwilling to refrain from benzodiazepines or alcohol during treatment. Participants were required to be MRI compatible and not have a neurological illness. After exclusions, the baseline sample consisted of 31 OCD patients and 28 healthy controls, while 24 patients and 17 controls were included in longitudinal analyses including the day before treatment, after one week (directly after treatment), and after three months (See Supplemental Fig. 1 for a flowchart with reasons for exclusions). The study was approved by the Regional Ethics Committee for South-Eastern Norway (2015/936) and all participants provided informed written consent.

**Table 1:** Baseline demographic and clinical characteristics.

	OCD (n = 31)	HC (n = 26)		
	M (SD)	M (SD)	t	p
Age	30.19 (9.21)	31 (10.73)	0.31	0.76
Education (years)	14.58 (2.41)	14.43 (2.30)	0.25	0.81
	n (%)	n (%)	χ^2^	P
Female	19 (61)	18 (64)	0.06	1
Handedness (right)	29 (94)	26 (93)	0.01	1
On medication	7 (23)	–	–	–
SSRI	6 (19)	–	–	–
Methylphenidate	1 (3)	–	–	–
Childhood onset of OCD	14 (45)	–	–	–
Major Depressive Disorder	9 (29)	–	–	–
Generalized Anxiety Disorder	9 (29)	–	–	–
Social Anxiety Disorder	7 (23)	–	–	–
Specific Phobia	4 (13)	–	–	–
Panic disorder with/without agoraphobia	3 (10)	–	–	–
Hypochondriasis	3 (10)	–	–	–
Dysthymia	2 (7)	–	–	–
Post-Traumatic Stress Disorder	1 (3)	–	–	–
Attention Deficit Hyperactivity Disorder	1 (3)	–	–	–
Somatization disorder	1 (3)	–	–	–
Pain disorder	1 (3)	–	–	–

Abbreviations: HC, healthy controls; OCD, obsessive–compulsive disorder; SSRI, selective serotonin reuptake inhibitors.

### 2.2 Measures

All participants were diagnosed using the Structured Clinical Interview (SCID) for DSM-IV (First et al., 2002), and healthy controls were free of any current or lifetime disorders. The Y-BOCS (Goodman et al., 1989), Patient Health Questionnaire (PHQ-9) (Kroenke et al., 2001), and Generalized Anxiety Disorder (GAD-7) (Spitzer et al., 2006) were used to measure severity of obsessive–compulsive, depressive, and anxiety symptoms. Clinical remission was defined as a total Y-BOCS score under 13 and response as a minimum of 35% reduction on the Y-BOCS (Mataix-Cols et al., 2016). The Behavior Rating Inventory of Executive Function (BRIEF) (Roth et al., 2013) was used to measure subjective problems in executive function in OCD patients and through informant reports from family members before treatment.

### 2.3 Bergen 4-Day treatment

The Bergen 4-Day Treatment (B4DT) format is delivered during four consecutive days in groups for 3–6 patients with a 1:1 ratio between patients and therapists (Havnen et al., 2014, Havnen et al., 2017, Launes et al., 2019, Riise et al., 2016, Riise et al., 2018). Routine clinical data and a randomized control trial have found a remission rate of 75%, while an additional 10% were improved, and 15% showed no significant change one week after treatment (Havnen et al., 2014, Havnen et al., 2017, Launes et al., 2019), which are largely maintained after four years (Hansen et al., 2019). The first day of the B4DT consists of a group session with psychoeducation and planning of individual exposure tasks. The next two days consist of therapist-assisted exposure with response prevention in relevant settings. Patients are also instructed to perform exposure between the second and fourth day. The last day consists of relapse prevention and planning of self-exposure for the next three weeks (Kvale et al., 2018).

### 2.4 Stop signal task

The SST (de Wit et al., 2012) required responding to the direction of an arrow (left or right) by pressing a button with the index finger of the concordant hand during go-trials (Supplemental Fig. 2). Participants were instructed to respond as quickly and accurately as possible. Go-trials were pseudo-randomly mixed with stop-trials where participants are instructed to withhold their response when a cross was overlaid on the arrow with a variable delay. The delay of the stop signal was continuously adapted by a staircase tracking mechanism, so that the participant reaches around 50% accuracy on stop-trials. Stop signal reaction time (SSRT) was calculated using the integration method over four blocks (Verbruggen et al., 2013). Exclusion criteria were go-trial error percentage over 40% or failed stop-trials outside of the 25–75% range (Congdon et al., 2012). Group differences in performance was tested using t-tests and repeated-measures ANOVAs (RM-ANOVA) including the two groups over the three time points.

### 2.5 Image acquisition and analyses

MRI was done on a 3 T General Electric Discovery MR750 with an eight-channel head coil at Haukeland university hospital, Bergen, Norway. An anatomical T1-weighted image was recorded in a 256 × 256 matrix, 192 slices, voxel size approximately isotropic 1 mm^3^, TE = 30 ms, TR = 7000 s, flip angle = 12°, FoV = 256 mm. We acquired 430 echo planar images using a 64x64 matrix, 34 slices (2.8 mm thickness with 0.2 mm gap), TR = 2100 ms, TE = 30 ms, flip angle = 80°, FoV = 22 mm, voxel size = 3.44 × 3.44 × 3 mm, interleaved slice excitation. Functional data were preprocessed in SPM12 using slice time correction, realignment, coregistering anatomical and functional images, normalization to MNI using unified segmentation, reslicing voxels to 3 mm^3^, and 8 mm smoothing with a FWHM kernel. Participants with movement exceeding one voxel were excluded. Accurate go-trials, accurate stop-trials, failed stop-trials were then modeled as 0 s events in subject-level models, along with six motion parameters. A high pass filter with 128 s cut-off was then applied to remove low frequency noise.

We used the generalized psychophysiological interaction toolbox (gPPI) (McLaren et al., 2012) to model task-related functional connectivity during accurate go-trials, accurate stop-trials, failed stop-trials. The left (MNI -23,-2,-16) and right amygdala (MNI 23,0,-16) were defined as spherical 5 mm seed regions based on the Anatomical Labeling Atlas as previous studies have found aberrant amygdala connectivity during tasks probing action cancellation and working memory in OCD patients and their unaffected siblings (de Vries et al., 2014, van Velzen et al., 2015). PPI models included the three task regressors, three PPI regressors, time course of the seed region, and six motion parameters.

Group comparisons for both activation and connectivity were done by entering successful response inhibition (SucStop > SucGo) and failed inhibition (FailStop > SucStop) into separate second-level models. We used permutation-based statistics for imaging analyses, as they are less dependent on statistical assumptions (Winkler et al., 2014). The Statistical Nonparametric Mapping (SnPM) toolbox (http://nisox.org/Software/SnPM13/) was used for baseline t-tests, and the Multivariate and Repeated Measures (MRM) toolbox (McFarquhar et al., 2016) for RM-ANOVAs.

We defined regions of interest (ROI) based on the findings of a recent meta-analysis of the SST (Cieslik et al., 2015). To ensure optimal placement we first investigated the effect of successful and failed inhibition across the whole sample at baseline (N = 59, voxel-wise p_FWE_ < 0.05), and then placed 10 mm spheres in the bilateral anterior insula/IFG, pre-SMA, operculum, inferior parietal cortex, and midline posterior cingulate cortex for successful inhibition (Table 2 and Supplemental Results). We excluded two healthy controls due to signal loss in the IFG. We did not find an effect of inhibition in the thalamus, dACC or subthalamic nucleus and these ROIs were therefore excluded. For failed inhibition we defined the midline dACC and pre-SMA (Table 2 and Supplemental Results). ROIs were combined into two separate binary masks for successful and failed inhibition, respectively. ROI analyses were performed by limiting the included voxels to the binary mask, which provided a single volume correction including all ROIs. Statistical significance was set at voxel-wise p_FWE_ < 0.05. Exploratory analyses at uncorrected p < .001 are also presented for comparisons with previous studies. Results of whole-brain analyses at an uncorrected threshold are presented in the Supplement.

**Table 2:** Regions of interest for successful and failed inhibition (defined as 10 mm spheres around peak).

Region	Hemisphere	MNI coordinates (X, Y, Z)
*Successful inhibition (SucStop > SucGo)*		
Anterior insula/IFG	L	–33, 23, 5
Anterior insula/IFG	R	35, 23, −11
Pre-SMA	L	−6, 11, 50
Pre-SMA	R	3, 20, 53
Operculum	L	−42, 5, 29
Operculum	R	44, 10, 29
Inferior parietal cortex	L	−55, −46, 35
Inferior parietal cortex	R	55, −46, 21
Posterior cingulate cortex	Midline	2, −24, 32
*Failed inhibition (FailStop > SucStop)*		
Dorsal ACC	Midline	0, 21, 34
Pre-SMA	Midline	0, 11, 62

Abbreviations: ACC, anterior cingulate cortex; IFG, inferior frontal gyrus; MNI, Montreal Neurological Institute; pre-SMA, pre-supplementary motor area.

## 3 Results

### 3.1 Demographics, symptoms, and executive problems

OCD patients and healthy controls were matched on age, gender and, educational status (Table 1). OCD patients showed significant improvements over time on the Y-BOCS (F(2, 46) = 112.07, p < .001) and GAD-7 (F(2, 36) = 12.71, p < .001), but not PHQ-9 (F(2, 34) = 1.37, p = .27). Healthy controls showed no significant changes in GAD-7 or PHQ-9 over time (Table 3). One week after treatment, 16 (67%) patients were in remission, six (25%) significantly improved, and two (8%) were unchanged. After three months, 19 (79%) were in remission, two (8%) significantly improved, and three (13%) unchanged. Based on the BRIEF, OCD patients reported worse problems in most executive domains than the normal population, particularly in task shifting and initiation. This was also supported by informant reports from the family of the patient (Supplemental Table 1).

**Table 3:** Symptom scores over time in OCD patients (n = 24) and healthy controls (n = 17).

Variable	Group	Before treatmentM (SD)	After one weekM (SD)	After three monthsM (SD)
Y-BOCS	OCD	26.83 (4.26)	10.33 (5.58)	10.33 (6.37)
GAD-7	OCD	12.46 (5.33)	8.41 (4.23)	7.10 (4.52)
	HC	2.16 (2.63)	1.89 (2.05)	2.00 (2.17)
PHQ-9	OCD	11.08 (5.83)	8.73 (6.10)	8.32 (5.49)
	HC	2.53 (1.71)	2.32 (1.83)	2.11 (1.53)

Abbreviations: GAD-7, Generalized Anxiety Disorder 7; HC, healthy controls; OCD, obsessive–compulsive disorder; PHQ-9, Patient Health Questionnaire 9; Y-BOCS, Yale-Brown Obsessive Compulsive Scale.

### 3.2 Task performance

There were no significant group differences in SSRT or go-trial reaction time at any time point (Table 4). The RM-ANOVA of SSRT showed no significant effects of group (F(1, 41) = 0.59, p = .44), time (F(1,82) = 0.06, p = .81), and group × time interaction (F(2,82) = 1.03, p = .36).

**Table 4:** Task performance inhibition between OCD (n = 24) and HC (n = 19) over time.

	Before treatmentM (SD)					After one weekM (SD)					After three monthsM (SD)			
	OCD	HC	t	p		OCD	HC	t	p		OCD	HC	t	p
SSRT (ms)	203.94 (28.18)	203.68 (47.09)	0.02	0.98		193.4 (35.80)	201.65 (55.39)	0.08	0.56		196.87 (33.81)	213.72 (48.19)	1.35	0.19
Mean go-trial RT (ms)	506.67 (103.38)	549.15 (141.47)	1.14	0.26		504.08 (120.81)	563.67 (187.45)	1.26	0.21		496.46 (101.87)	554.5 (174.87)	1.36	0.18
Errors on go-trials (%)	0.02 (0.01)	0.01 (0.02)	0.37	0.71		0.02 (0.02)	0.02 (0.02)	0.37	0.52		0.02 (0.03)	0.03 (0.04)	1.46	0.15

Abbreviations: HC, healthy controls; OCD, obsessive–compulsive disorder; RT, response time.

### 3.3 Task-related brain activation

**Fig. 1:**
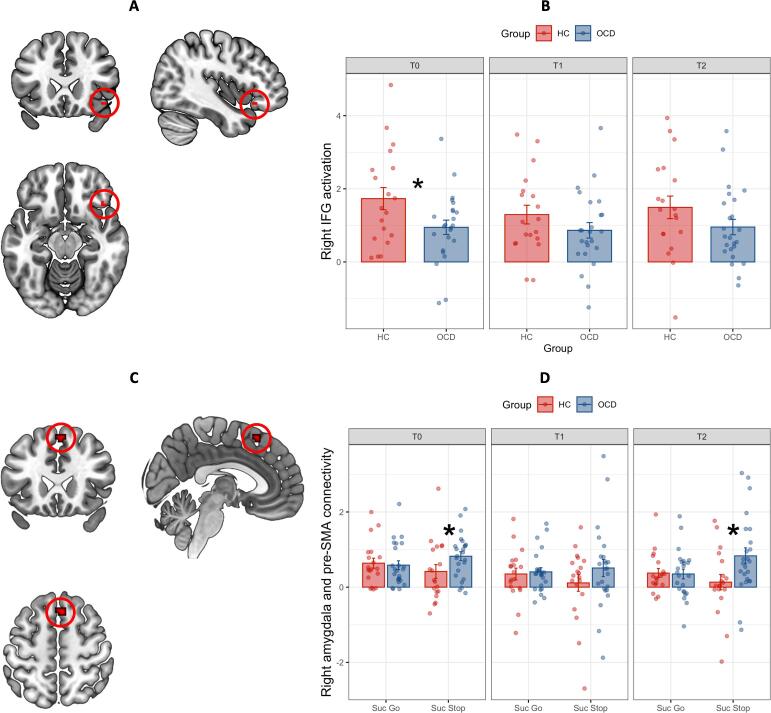
Right IFG activation and connectivity between the right amygdala and right pre-SMA during successful inhibition in OCD patients (n = 24) and healthy controls (n = 17). Legend Fig. 1: Panel A depicts the voxels in the right inferior frontal gyrus (IFG) where OCD patients showed less activation than healthy controls during successful inhibition compared to successful go-trials. B shows activation parameter estimates in the right IFG during successful inhibition compared to successful go-trials for each group and timepoint, as well as individual data points. Panel C depicts the voxels in the pre-SMA where OCD patients and healthy controls showed significant differences in right amygdala connectivity during successful inhibition versus successful go-trials D shows condition-specific connectivity estimates between the right amygdala and right pre-SMA during successful stop and go-trials. Healthy controls show a non-significant tendency towards stronger connectivity between the right pre-SMA and right amygdala during successful go-trials, while OCD patients show significantly stronger connectivity between the two regions during successful inhibition before treatment and three months after treatment. * indicates a significant group difference at p < .05 based on parameter estimates extracted using 6 mm spheres, with error bars representing one standard error.

During successful inhibition, OCD patients (n = 31) showed less activation than healthy controls (n = 26) in the right IFG before treatment (Table 5, Fig. 1). This right IFG hypoactivation in OCD patients was not found in the RM-ANOVA, which showed no significant effects of group, time or group × time interaction (OCD n = 24 versus HC n = 17). Right IFG hypoactivation in OCD patients before treatment was also evident in whole-brain analyses at an uncorrected threshold (Supplemental Table 6).

**Table 5:** Group differences in activation during inhibition between OCD (n = 31) and HC (n = 26) before treatment.

**Region**	**Side**	**BA**	**Voxels**	**X**	**Y**	**Z**	**T**	**p_FWE_**	**p_Unc_**	**Direction**
IFG	R	47	3	42	20	−16	3.76	0.023	0.001	HC > OCD

Abbreviations: HC, healthy controls; IFG, inferior frontal gyrus; OCD, obsessive–compulsive disorder; R, right.

During error processing, OCD patients (n = 24) showed less pre-SMA activation than healthy controls (n = 26) (MNI 0,14,62, t = 2.98, p_FWE_ = 0.05) before treatment. The RM-ANOVA showed no significant effects of group, time or group × time interaction (OCD n = 24 versus HC n = 17). Pre-SMA hypoactivation in OCD patients was not significant in exploratory whole-brain analysis (Supplemental Table 7).

### 3.4 Task-related connectivity

**Fig. 2:**
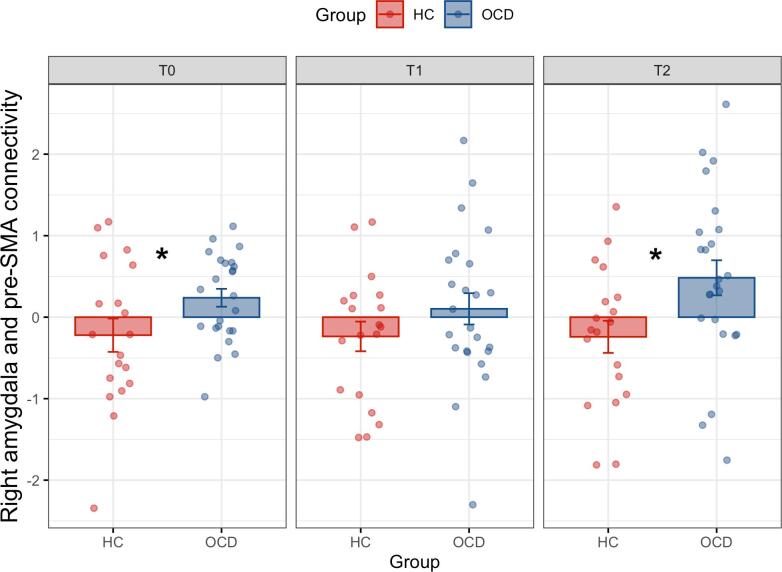
Task-related connectivity between the right amygdala and right pre-SMA during successful inhibition in OCD patients (n = 24) and healthy controls (n = 17). Psychophysiological interaction for connectivity between the right amygdala and right pre-SMA during successful stop and go-trials. Healthy controls show no significant changes in connectivity between the successful inhibition and successful go-trials, while OCD patients show significantly stronger more positive connectivity during successful inhibition than successful go-trials before treatment and three months after treatment. * indicates a significant group difference at p < .05 based on parameter estimates extracted using 6 mm spheres, with error bars representing one standard error.

Before treatment, OCD patients (n = 31) compared to controls (n = 26) showed significantly more connectivity between the right amygdala and pre-SMA during successful inhibition, while more connectivity with the right IFG reached a trend level (Table 6). Right amygdala-pre-SMA hyperconnectivity was also found at a corrected threshold in whole-brain analysis (Supplemental Table 8). There were no significant group differences for left amygdala connectivity in ROI analyses, but some findings emerged in exploratory whole-brain analyses (Supplemental Table 9). To investigate the group differences over time for the right amygdala we ran a 2x3 RM-ANOVA (24 OCD versus 17 HC over three time points), which revealed a significant effect of group in the right pre-SMA, with no significant effects of time or group × time interaction (Table 7, Fig. 1). This was also evident in whole-brain analysis at an uncorrected threshold (Supplemental Table 12). Within-group analyses of extracted beta values found that OCD patients showed increased positive connectivity between the right amygdala and the right IFG for successful inhibition versus go-trials (t(30) = 2.37, p = .02), while healthy controls showed no significant difference between task conditions (t(25) = -1.78, p = .09, Fig. 2). For connectivity between amygdala and pre-SMA, 2x3 RM-ANOVAs (successful stop- versus successful go over three time points) found that OCD patients showed increased connectivity during successful inhibition versus go-trials (F(1,23) = 9.89, p < .01, η^2^_p_ = .30). In comparison, healthy controls showed decreased connectivity during successful inhibition versus go-trials (F(1,16) = 6.68, p = .02, η^2^_p_ = .29).

**Table 6:** Group differences in amygdala connectivity during inhibition between OCD (n = 31) and HC (n = 26) before treatment.

**Seed region**	**Region**	**Side**	**BA**	**Voxels**	**X**	**Y**	**Z**	**T**	**p_FWE_**	**p_Unc_**	**Direction**
R amygdala	Pre-SMA	Midline	8	14	0	26	59	5.21	0.001	<0.001	OCD > HC
R amygdala	IFG	R	47	10	33	26	−16	3.46	0.071	<0.001	OCD > HC

Abbreviations: HC, healthy controls; IFG, inferior frontal gyrus; OCD, obsessive–compulsive disorder; Pre-SMA, pre-supplementary motor area; R, right.

**Table 7:** Effect of group for amygdala connectivity during inhibition between OCD (n = 24) and HC (n = 17) over time.

**Seed region**	**Region**	**Side**	**BA**	**Voxels**	**X**	**Y**	**Z**	**F**	**p_FWE_**	**p_Unc_**	**Direction**
R amygdala	Pre-SMA	R	8	2	3	23	56	17.73	0.042	<0.001	OCD > HC

Abbreviations: HC, healthy controls; OCD, obsessive–compulsive disorder; Pre-SMA, pre-supplementary motor area; R, right.

On the failed inhibition contrast, there were no group differences in connectivity with the left or right amygdala before treatment. RM-ANOVAs also found no significant group, time, or group × time interaction effects.

### 3.5 Correlations of task performance and imaging with behavioral and clinical measures

**Supplementary figure 5:**
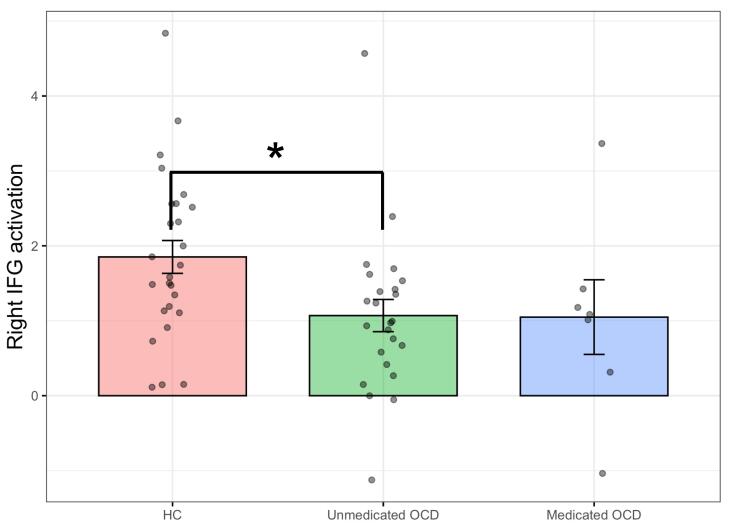


**Supplementary figure 6:**
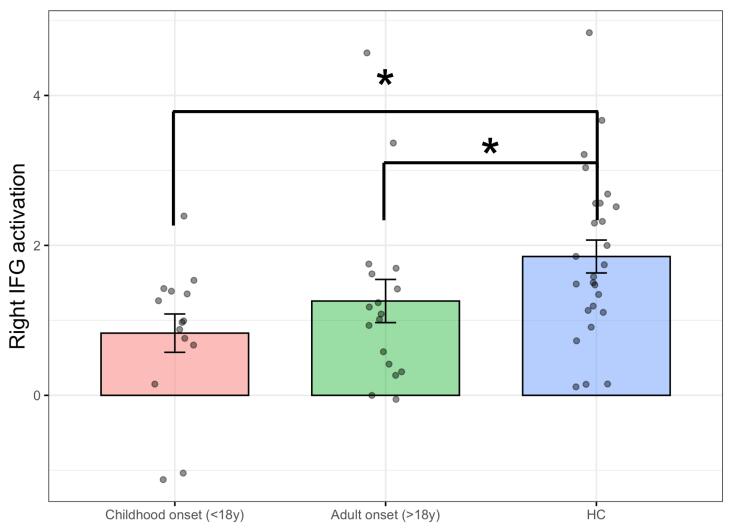


**Supplementary figure 7:**
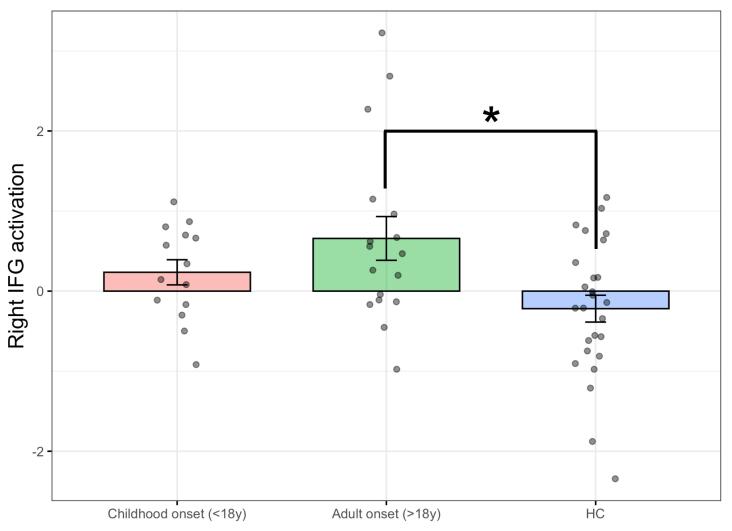


We used MarsBar to extract the mean estimate of IFG activation, amygdala-pre-SMA, and amygdala-IFG connectivity using 6 mm spheres around the peak voxel. Peak voxels for pre-treatment IFG activation and amygdala-IFG connectivity was defined as the voxel with the highest t-value in the *t*-test comparing OCD patients and healthy controls (Table 5, Table 6), while the peak voxel for amygdala-pre-SMA connectivity was defined as the voxel with the highest F-value in the effect of group in the repeated-measures ANOVA (Table 7). Exploratory tests in IBM SPSS Statistics 25 were then used to assess the relationship between these variables and symptom severity or task performance. Using Pearson correlations we found no significant relations between pre-treatment activation or connectivity and pre-treatment Y-BOCS, GAD-7 or PHQ-9 severity. A multilevel regression analysis including all time points found that more right amygdala-IFG connectivity during successful inhibition was related to longer SSRT in OCD patients (b = 7.39, SE = 3.45, t = 2.14, p = .037), which was not found for right amygdala-pre-SMA connectivity. Linear regression models were used to investigate if pre-treatment SSRT, IFG activation, amygdala-pre-SMA, or amygdala-IFG connectivity predicted Y-BOCS severity after treatment while adjusting for baseline Y-BOCS severity, but these models did not result in any significant findings. Finally, two-sample t-tests suggested that medication use, age of onset, or comorbid anxiety or mood disorders were not significantly related to IFG activation, amygdala-pre-SMA, or amygdala-IFG connectivity in OCD patients (Supplementary Figs. 5–8).

## 4 Discussion

We compared OCD patients and healthy controls on task performance, task-related brain activation, and connectivity during response inhibition and error processing, and studied how these measures changed after effective behavioral treatment. As hypothesized, OCD patients compared to controls showed less right IFG activation during successful response inhibition before treatment, but there were no significant changes over time. During successful inhibition, patients also showed more connectivity between the right amygdala and the pre-SMA across time, and with right IFG before treatment, whereas healthy controls showed no significant difference in connectivity between go- and stop-trials. We did not observe a difference in task performance between patients and controls before or after treatment, but exploratory findings suggested that IFG-amygdala connectivity correlated positively with SSRT in patients across time. Contrary to our hypotheses, patients showed no significant changes in IFG activation or fronto-limbic connectivity after treatment, suggesting that these are stable vulnerability markers of OCD that are unrelated to treatment outcome.

Norman and colleagues (Norman et al., 2019) have suggested that key regions for inhibitory control are underrecruited in OCD, leading to worse inhibitory control. They further propose that patients are aware of this underperformance, leading to an increased error response. In turn, this leads to greater limbic involvement and even poorer inhibitory control (Norman et al., 2019). The present findings of less task-related IFG activation and more fronto-limbic connectivity partially support these hypotheses. However, we did not see worse task performance during inhibition nor more activation during error processing. Norman and colleagues (Norman et al., 2019) only found a very small difference in response time between OCD patients and healthy controls suggesting that very large samples are needed to reliably detect this behavioral difference. Further, a combined EEG-fMRI study found that OCD patients compared with healthy controls can show stronger error-related negativity without a significant difference in pre-SMA BOLD amplitude (Grutzmann et al., 2016).

The finding of IFG hypoactivation in OCD patients versus healthy controls replicates the finding of our earlier cross-sectional study using this same stop-signal paradigm in a different sample, scanner, and country (de Wit et al., 2012). It is also in line with structural studies showed altered volume of this region in OCD (de Wit et al., 2014) and a negative association between IFG volume and inhibitory performance (Menzies et al., 2007). Hypoactivation of the IFG in OCD patients could be related to subtle alterations in attention to the stop-signal, response selection or inhibitory impairment, since the IFG plays an integral role in these processes (Aron et al., 2014). Although we did not observe any differences in task performance at the group-level, we did find that increased connectivity between the right amygdala and right IFG during successful inhibition was related to longer SSRT in OCD, suggestive of limbic interference on inhibitory control (de Vries et al., 2014, van Velzen et al., 2015).

The increased amygdala-pre-SMA connectivity during successful inhibition versus go-trials in patients may be indicative of increased salience of the stop-signal in general, possibly due to an oddball phenomenon (Dayan-Riva et al., 2019). Interestingly, fronto-limbic connectivity in the same sample of OCD patients was reduced after treatment when measured at rest (Thorsen et al., 2020). This suggests that limbic interference may be modulated by increased task demands (de Vries et al., 2014). Future work could use tasks with varying task loads and emotional stimuli to better understand if abnormal task performance or network activation are a result of altered cognitive capacity, emotional interference, or inflexibility (Bradbury et al., 2011, Thorsen et al., 2018).

Contrary to our previous reports, we did not replicate findings of increased pre-SMA activation (de Wit et al., 2012) or increased connectivity between the left IFG and the amygdala in patients versus controls (van Velzen et al., 2015). These null findings could be influenced by sample size and sample characteristics. Moreover, we here observed more positive fronto-limbic connectivity during successful inhibition in OCD compared to controls, whereas we previously found increased negative connectivity during inhibition, but positive connectivity during working memory (de Vries et al., 2014). We cannot exclude the possibility that differences in direction of fronto-limbic connectivity alterations are related to methodological differences in PPI analyses. Less pre-SMA activation during failed inhibition versus successful stop trials in OCD patients compared to healthy controls before treatment was also an unexpected finding. A recent *meta*-analysis found that OCD patients on average showed more pre-SMA activation during error processing than healthy controls (Norman et al., 2019), but this was not found in either of the two included studies using the SST (de Wit et al., 2012, Rubia et al., 2010). It is unclear if the correlates of error processing differ by the kind of response inhibition. The SST specifically measures action cancellation, as the stop signal is presented after the presentation of the go-signal (van Velzen et al., 2014). Action cancellation may be a more difficult form of response inhibition and may be particularly relevant for OCD as these patients show difficulties in stopping compulsions that have already been initiated (van Velzen et al., 2014). Parametric studies of working memory and planning have suggested that OCD patients are unable to maintain the required increases in activation during more difficult trials when compared to healthy controls (de Vries et al., 2014, Heinzel et al., 2018, van den Heuvel et al., 2005). It is therefore possible that OCD patients are similarly unable to maintain pre-SMA activation during error processing in the more difficult SST than other response inhibition tasks.

This study is limited by the sample size. Thus, most findings from ROI analyses were only significant at an uncorrected threshold in whole-brain analyses. The limited sample size also precluded equivalence tests to formally determine that activation and connectivity estimates were equal over time in OCD patients. A wait-list control condition may also have formally excluded the possibility of non-specific variation over time.

This is the first study to show that IFG activation and fronto-limbic connectivity during response inhibition in OCD does not change after concentrated ERP. Furthermore, we found no evidence that pre-treatment IFG activation or fronto-limbic connectivity predicted treatment outcome. The present findings extend previous evidence that both OCD patients and unaffected siblings (de Wit et al., 2012, van Velzen et al., 2015), compared to unrelated healthy controls, show abnormal IFG activation and fronto-limbic connectivity during response inhibition. Together with the lack of change over time as presented in this study, this suggests that these are trait markers of OCD.

## 5 Previous presentations

A preprint has been deposited at the Open Science Foundation (https://osf.io/achuw).

## 6 Disclosures

OAvdH has received speaker’s honorarium from Benecke.

## CRediT authorship contribution statement

**Anders Lillevik Thorsen:** Conceptualization, Software, Formal analysis, Investigation, Data curation, Writing - original draft, Writing - review & editing, Visualization, Project administration. **Stella J. de Wit:** Conceptualization, Methodology, Software, Validation, Writing - original draft, Writing - review & editing, Supervision. **Pernille Hagland:** Investigation, Data curation, Writing - original draft, Writing - review & editing. **Olga Therese Ousdal:** Conceptualization, Writing - original draft, Writing - review & editing, Supervision. **Bjarne Hansen:** Conceptualization, Writing - original draft, Writing - review & editing, Supervision, Project administration. **Kristen Hagen:** Investigation, Supervision, Writing - original draft, Writing - review & editing, Supervision, Project administration. **Gerd Kvale:** Conceptualization, Supervision, Writing - original draft, Writing - review & editing, Supervision, Project administration, Funding acquisition. **Odile A. van den Heuvel:** Conceptualization, Methodology, Validation, Writing - original draft, Writing - review & editing, Supervision, Project administration.

## Declaration of Competing Interest

The authors declare that they have no known competing financial interests or personal relationships that could have appeared to influence the work reported in this paper.

## Figures and Tables

**Supplementary figure 8:**