# Supplementary material for: Stable inhibition-related inferior frontal hypoactivation and fronto-limbic hyperconnectivity in obsessive–compulsive disorder after concentrated exposure therapy
Source: Neuroimage Clin. 2020 Oct 13;28:102460. doi: 10.1016/j.nicl.2020.102460 (PMC7606869; doi:10.1016/j.nicl.2020.102460)
Supplement: Supplementary data 1 [file mmc1.docx]

**Supplemental materials**

**Supplemental results**

***Task effects***

A series of one-sample t-tests thresholded at pFWE<.05 over the whole brain were performed to investigate effects of successful and failed inhibition, respectively. A one-sample t-test across OCD patients and healthy controls (HC) found significant activation in the ROI for both successful and failed inhibition (Table 1). Separate t-tests per group showed that both groups showed significant activation in the bilateral IFG/anterior insula, right operculum and right inferior parietal cortex for successful inhibition. Only HC showed significant activation in the bilateral pre-SMA, posterior cingulate cortex, and left inferior parietal cortex. No group showed significant activation in the left operculum (Supplemental Table 2 and 3). Only HC showed significant activation in the pre-SMA and dACC during failed inhibition (Supplemental Table 4 and 5).

**Supplemental Tables**

Supplemental Table 1 *Behavior Rating Inventory of Executive Function from OCD patients (n=26) and informants (n=23) relative to population norms**

|  | Patient | | | | Informant | | | |
| --- | --- | --- | --- | --- | --- | --- | --- | --- |
| Variable | M | t | p | d** | M | t | p | d** |
| Initiating | 65.54 | 5.29 | .00 | 1.02 | 60.91 | 3.97 | .00 | 0.81 |
| Shifting | 62.19 | 5.04 | .00 | 0.97 | 58.13 | 3.15 | .00 | 0.64 |
| Working Memory | 63.12 | 4.34 | .00 | 0.84 | 58.30 | 2.51 | .02 | 0.51 |
| Metacognition Index | 61.35 | 4.09 | .00 | 0.79 | 55.35 | 2.06 | .05 | 0.42 |
| Emotional Control | 61.85 | 4.00 | .00 | 0.77 | 57.65 | 3.51 | .00 | 0.72 |
| Planning and Organizing | 60.85 | 3.95 | .00 | 0.76 | 50.61 | 1.08 | .29 | 0.22 |
| Global Executive Composite | 61.23 | 3.87 | .00 | 0.74 | 56.05 | 2.52 | .02 | 0.53 |
| Behavioral Regulation Index | 58.27 | 3.21 | .00 | 0.62 | 55.05 | 2.11 | .05 | 0.44 |
| Task Monitor | 58.62 | 2.82 | .01 | 0.54 | 51.78 | 0.59 | .56 | 0.12 |
| Inhibition | 55.35 | 1.66 | .11 | 0.32 | 51.87 | 0.31 | .76 | 0.06 |
| Organization of Materials | 51.38 | 0.38 | .71 | 0.07 | 50.61 | 0.11 | .92 | 0.02 |
| Self Monitoring | 47.77 | -1.56 | .13 | -0.30 | 47.55 | -1.50 | .15 | -0.31 |

*Scores were converted to t-scores according to age- and gender-adjusted norms. **calculated as Cohen’s d = t/√df. Abbreviations: HC, healthy controls; IFG, inferior frontal gyrus; OCD, obsessive-compulsive disorder; R, right.

Supplemental Table 2 *Whole-brain task effect of successful inhibition > successful go in OCD patients before treatment (n=31)*

| Region | Side | BA | Voxels | X | Y | Z | t | p_FWE_ | p_Unc_ |
| --- | --- | --- | --- | --- | --- | --- | --- | --- | --- |
| Middle occipital gyrus | R | 18/19/37/21/22 | 1356 | 33 | -85 | 8 | 10.51 | <.001 | <.001 |
| Inferior occipital gyrus | L | 18/19/37/21 | 971 | -45 | -70 | -4 | 9.90 | <.001 | <.001 |
| IFG/anterior insula | R | 47/13 | 229 | 33 | 23 | -7 | 8.30 | <.001 | <.001 |
| IFG/anterior insula | L | 47/13 | 126 | -33 | 26 | -1 | 7.94 | <.001 | <.001 |
| Middle temporal gyrus | R | 21 | 41 | 51 | -28 | -4 | 6.87 | <.001 | <.001 |
| Precentral gyrus | R | 48/44/6 | 159 | 48 | 14 | 29 | 6.73 | <.001 | <.001 |
| Inferior parietal lobule | L | 40 | 9 | -33 | -46 | 38 | 5.01 | .015 | <.001 |
| Ventrolateral PFC | R | 46 | 1 | 48 | 32 | 17 | 4.66 | .045 | <.001 |

Abbreviations: BA, Brodmann area; FWE, family-wise error; IFG, inferior frontal gyrus; L, left; PFC, prefrontal cortex; R, right; Unc, uncorrected.

Supplemental Table 3 *Whole-brain task effect of successful inhibition > successful inhibition in HC before treatment (n=26)*

| Region | Side | BA | Voxels | X | Y | Z | t | p_FWE_ | p_Unc_ |
| --- | --- | --- | --- | --- | --- | --- | --- | --- | --- |
| IFG/anterior insula | R | 47/13/44/9 | 707 | 45 | 20 | -7 | 9.91 | <.001 | <.001 |
| Middle occipital gyrus | L | 19/18/37/20 | 817 | -48 | -76 | -1 | 9.68 | <.001 | <.001 |
| Fusiform/temporal gyri | R | 37/18/19/7 | 1753 | 42 | -58 | -13 | 8.64 | <.001 | <.001 |
| IFG/anterior insula | L | 47/13 | 209 | -33 | 20 | 5 | 8.01 | <.001 | <.001 |
| Dorsolateral PFC | R | 9/ | 42 | 42 | 47 | 20 | 5.78 | .003 | <.001 |
| ACC | R | 32 | 79 | 6 | 29 | 35 | 5.74 | .003 | <.001 |
| Angular gyrus | L | 40/42/22 | 40 | -57 | -52 | 35 | 5.67 | .004 | <.001 |
| Posterior cingulate gyrus | Midline | 23 | 7 | 0 | -28 | 26 | 5.46 | .008 | <.001 |
| Amygdala | R | 34 | 4 | 27 | 5 | -19 | 5.17 | .012 | <.001 |
| Precuneus | L | 7 | 5 | -12 | -70 | 35 | 5.16 | .013 | <.001 |
| Middle temporal gyrus | L | 21 | 9 | -54 | -28 | -4 | 5.12 | .014 | <.001 |
| Pre-SMA | R | 6 | 5 | 15 | 11 | 65 | 5.11 | .015 | <.001 |
| Dorsolateral PFC | R | 46 | 11 | 30 | 53 | 23 | 4.89 | .025 | <.001 |
| Calcarine sulcus | L | 18 | 3 | -15 | -76 | 8 | 4.89 | .026 | <.001 |
| Calcarine sulcus/cuneus | R | 18/17 | 12 | 6 | -94 | 11 | 4.82 | .030 | <.001 |
| WM | Midline |  | 1 | 0 | -22 | -1 | 4.76 | .035 | <.001 |
| Hippocampus | R | 34 | 1 | 18 | -4 | -13 | 4.71 | .039 | <.001 |
| Superior parietal lobule | L | 7 | 1 | -27 | -52 | 44 | 4.70 | .041 | <.001 |

Abbreviations: ACC, anterior cingulate cortex; BA, Brodmann area; FWE, family-wise error; IFG, inferior frontal gyrus; L, left; PFC, prefrontal cortex; R, right; SMA, supplementary motor area; Unc, uncorrected; WM, white matter.

Supplemental Table 4 *Whole-brain task effect of failed inhibition in OCD patients before treatment (n=31)*

| Region | Side | BA | Voxels | X | Y | Z | t | p_FWE_ | p_Unc_ |
| --- | --- | --- | --- | --- | --- | --- | --- | --- | --- |
| Temporal pole | L | 22 | 25 | -57 | 8 | -1 | 5.83 | .001 | <.001 |
| Calcarine sulcus/lingual gyrus | R | 17/19 | 39 | 21 | -61 | 5 | 5.25 | .005 | <.001 |
| Calcarine sulcus/lingual gyrus | L | 17/19 | 20 | -18 | -64 | 5 | 5.00 | .014 | <.001 |
| Temporal pole | R | 38 | 5 | 33 | 11 | -25 | 4.61 | .037 | <.001 |
| Precentral gyrus | L | 6/48 | 1 | -57 | 5 | 20 | 4.59 | .038 | <.001 |
| WM | Bilateral |  | 1 | 0 | -25 | -4 | 4.57 | .040 | <.001 |

Abbreviations: BA, Brodmann area; FWE, family-wise error; L, left; R, right; Unc, uncorrected; WM, white matter.

Supplemental Table 5 *Whole-brain task effect of failed inhibition in HC before treatment (n=26)*

| Region | Side | BA | Voxels | X | Y | Z | t | p_FWE_ | p_Unc_ |
| --- | --- | --- | --- | --- | --- | --- | --- | --- | --- |
| Pre-SMA | Bilateral | 6 | 132 | 0 | 11 | 62 | 7.09 | <.001 | <.001 |
| Lingual gyrus | L | 19/18 | 57 | -21 | -58 | 2 | 5.92 | .001 | <.001 |
| Lingual gyrus | R | 19 | 40 | 24 | -55 | 2 | 5.91 | .001 | <.001 |
| Precentral gyrus | R | 4 | 79 | 48 | -10 | 56 | 5.57 | .003 | <.001 |
| Postcentral gyrus | L | 3 | 21 | -45 | -16 | 56 | 5.23 | .008 | <.001 |
| Middle cingulate gyrus | Bilateral | 23 | 12 | 0 | -13 | 41 | 5.16 | .009 | <.001 |
| Hippocampus (tail) | L | 27 | 3 | -15 | -28 | -7 | 5.09 | .010 | <.001 |
| Temporal pole | L | 38 | 3 | -51 | 14 | -7 | 4.77 | .028 | <.001 |
| ACC | Bilateral | 24 | 5 | 0 | 14 | 41 | 4.77 | .029 | <.001 |
| Precentral gyrus | R | 4 | 3 | 36 | -25 | 68 | 4.75 | .030 | <.001 |
| Precentral gyrus | R | 6 | 1 | 27 | -25 | 71 | 4.73 | .033 | <.001 |
| Vermis | Bilateral | 27 | 2 | 0 | -40 | 2 | 4.65 | .042 | <.001 |

Abbreviations: ACC, anterior cingulate cortex; BA, Brodmann area; FWE, family-wise error; L, left; R, right; pre-SMA, pre-supplementary motor area; ACC, anterior cingulate cortex; Unc, uncorrected.

Supplemental Table 6 *Whole-brain group differences in activation during successful inhibition between OCD (n=31) and HC (n=26) before treatment*

| Region | Side | BA | Voxels | X | Y | Z | t | p_FWE_ | p_Unc_ | Direction |
| --- | --- | --- | --- | --- | --- | --- | --- | --- | --- | --- |
| Hippocampus | R | 34 | 3 | 18 | -4 | -16 | 3.86 | 0.213 | <.001 | HC>OCD |
| Middle cingulate | R | 23 | 12 | 12 | -4’9 | 38 | 3.85 | 0.216 | <.001 | HC>OCD |
| IFG | R | 47 | 2 | 42 | 20 | -16 | 3.83 | 0.226 | <.001 | HC>OCD |
| Precuneus | R | 7 | 1 | 0 | -61 | 53 | 3.43 | 0.463 | <.001 | HC>OCD |

Abbreviations: BA, Brodmann area; FWE, family-wise error; IFG, inferior frontal gyrus; L, left; R, right; Unc, uncorrected.

Supplemental Table 7 *Whole-brain group differences in activation during failed inhibition between OCD (n=31) and HC (n=26) before treatment*

| Region | Side | BA | Voxels | X | Y | Z | t | p_FWE_ | p_Unc_ | Direction |
| --- | --- | --- | --- | --- | --- | --- | --- | --- | --- | --- |
| dmPFC | L | 9/8 | 13 | -3 | 47 | 50 | 4.30 | 0.073 | <.001 | HC>OCD |
| Operculum | R | 44 | 4 | 33 | 11 | 32 | 3.55 | 0.341 | <.001 | HC>OCD |
| IFG | R | 47 | 5 | 42 | 41 | -7 | 3.09 | 0.648 | <.001 | HC>OCD |
| WM | L |  | 1 | -9 | -1 | 29 | 2.26 | 0.971 | <.001 | HC>OCD |

Abbreviations: BA, Brodmann area; FWE, family-wise error; dmPFC, dorsomedial prefrontal cortex; IFG, inferior frontal gyrus; L, left; R, right; Unc, uncorrected; WM, white matter.

Supplemental Table 8 *Group differences in right amygdala connectivity during successful inhibition between OCD (n=31) and HC (n=26) before treatment*

| Region | Side | BA | Voxels | X | Y | Z | t | p_FWE_ | p_Unc_ | Direction |
| --- | --- | --- | --- | --- | --- | --- | --- | --- | --- | --- |
| Pre-SMA | Bilateral | 8 | 27 | 0 | 26 | 59 | 4.93 | .014 | <.001 | OCD>HC |
| IFG | R | 47 | 6 | 33 | 26 | -16 | 3.46 | .535 | <.001 | OCD>HC |
| Putamen | R |  | 7 | 27 | -1 | -1 | 3.43 | .556 | <.001 | OCD>HC |
| Temporal pole | R | 21 | 5 | 54 | 11 | -22 | 3.31 | .637 | <.001 | OCD>HC |
| Medial PFC | R | 10 | 2 | 15 | 65 | 8 | 3.18 | .726 | <.001 | OCD>HC |
| Middle temporal gyrus | R | 37 | 3 | 45 | -64 | 8 | 3.15 | .748 | <.001 | OCD>HC |
| Middle occipital gyrus | L | 19 | 2 | -27 | -85 | 29 | 3.11 | .770 | <.001 | OCD>HC |
| Medial PFC | R | 10 | 1 | 21 | 62 | 8 | 2.71 | .949 | <.001 | OCD>HC |
| Middle occipital gyrus | L | 39 | 2 | -39 | -73 | 26 | 2.53 | .979 | <.001 | OCD>HC |
| Caudate nucleus | L | 25 | 1 | -9 | 14 | -4 | 2.04 | .999 | <.001 | OCD>HC |
| WM | L |  | 1 | -27 | -43 | 11 | 3.73 | .344 | <.001 | HC>OCD |
| Hippocampus (tail) | L | 37 | 3 | -36 | -31 | -4 | 3.43 | .552 | <.001 | HC>OCD |
| WM | L |  | 5 | -36 | -16 | 26 | 3.25 | .678 | <.001 | HC>OCD |
| Precuneus | L | 30 | 3 | -21 | -49 | 11 | 2.73 | .936 | <.001 | HC>OCD |

Abbreviations: BA, Brodmann area; FWE, family-wise error; IFG, inferior frontal gyrus; L, left; R, right; PFC, prefrontal cortex; pre-SMA, pre-supplementary motor area; Unc, uncorrected; WM, white matter.

Supplemental Table 9 *Group differences in left amygdala connectivity during successful inhibition between OCD (n=31) and HC (n=26) before treatment*

| Region | Side | BA | Voxels | X | Y | Z | t | p_FWE_ | p_Unc_ | Direction |
| --- | --- | --- | --- | --- | --- | --- | --- | --- | --- | --- |
| Calcarine sulcus | R | 18 | 9 | 21 | -61 | 20 | 3.95 | .211 | <.001 | OCD>HC |
| Medial PFC | R | 10 | 2 | 18 | 65 | 8 | 3.67 | .354 | <.001 | OCD>HC |
| Calcarine sulcus | L | 18 | 2 | -21 | -67 | 20 | 3.49 | .472 | <.001 | OCD>HC |
| IFG | R | 48 | 1 | 42 | 32 | 17 | 3.05 | .774 | <.001 | OCD>HC |
| WM | R |  | 1 | 27 | 14 | 38 | 3.03 | .788 | <.001 | OCD>HC |
| WM | R |  | 1 | 24 | 11 | 41 | 2.93 | .838 | <.001 | OCD>HC |
| WM | L |  | 1 | -30 | -34 | 35 | 2.47 | .971 | <.001 | OCD>HC |

Abbreviations: BA, Brodmann area; FWE, family-wise error; IFG, inferior frontal gyrus; L, left; PFC, prefrontal cortex; R, right; Unc, uncorrected; WM, white matter.

Supplemental Table 10 *Effects of group, time and group×time interaction for activation during successful inhibition in OCD (n=24) and HC (n=17)*

| Effect | Region | Side | BA | Voxels | X | Y | Z | F | p_FWE_ | p_Unc_ |
| --- | --- | --- | --- | --- | --- | --- | --- | --- | --- | --- |
| Group | WM |  |  |  |  |  |  |  |  | <.001 |
| Time | Middle occipital gyrus | L | 18 | 18 | -27 | -88 | 5 | 10.38 | .678 | <.001 |
| Time | WM | R |  | 3 | 30 | 5 | 41 | 9.29 | .719 | <.001 |
| Time | Paracentral lobule | R | 5 | 1 | 6 | -43 | 50 | 8.58 | .832 | <.001 |
| Time | Premotor cortex | R | 6 | 7 | 39 | 11 | 53 | 12.88 | .218 | <.001 |
| Interaction | WM | L |  | 2 | -48 | -40 | -7 | 10.64 | .489 | <.001 |
| Interaction | Parahippocampal gyrus | L | 37 | 5 | -30 | -34 | -10 | 11.84 | .317 | <.001 |
| Interaction | WM | L |  | 1 | -45 | -37 | -4 | 8.75 | .800 | <.001 |
| Interaction | Medial frontal gyrus | L | 10 | 1 | -6 | 65 | 11 | 7.71 | .933 | <.001 |
| Interaction | Inferior parietal lobule | L | 39 | 58 | -42 | -67 | 38 | 13.71 | .157 | <.001 |
| Interaction | WM | R |  | 3 | 42 | -46 | 23 | 9.34 | .710 | <.001 |
| Interaction | Angular gyrus | R | 39 | 30 | 48 | -67 | 32 | 11.94 | .304 | <.001 |
| Interaction | Medial frontal gyrus | L | 10 | 9 | -3 | 59 | 26 | 7.99 | .903 | <.001 |
| Interaction | Posterior cingulate gyrus | L | 23 | 61 | -9 | -46 | 32 | 13.05 | .200 | <.001 |
| Interaction | Middle cingulate gyrus | R | 23/24 | 23 | 12 | -4 | 41 | 11.33 | .387 | <.001 |
| Interaction | Superior frontal gyrus | R | 9 | 1 | 9 | 56 | 38 | 7.34 | .960 | <.001 |
| Interaction | Precuneus | L | 7 | 2 | 0 | -64 | 41 | 8.63 | .817 | <.001 |
| Interaction | Middle frontal gyrus | L | 8 | 1 | -33 | 23 | 50 | 8.58 | .825 | <.001 |

Abbreviations: BA, Brodmann area; FWE, family-wise error; L, left; R, right; Unc, uncorrected; WM, white matter.

Supplemental Table 11 *Effects of group, time and group×time interaction for activation during failed inhibition in OCD (n=24) and HC (n=17)*

| Effect | Region | Side | BA | Voxels | X | Y | Z | F | p_FWE_ | p_Unc_ |
| --- | --- | --- | --- | --- | --- | --- | --- | --- | --- | --- |
| Time | Thalamus | L |  | 2 | -3 | -4 | -1 | 7.56 | .931 | <.001 |
| Time | Superior occipital gyrus | L | 18 | 3 | -18 | -82 | 5 | 7.67 | .923 | <.001 |
| Time | Superior occipital gyrus | L | 18 | 3 | -21 | -79 | 8 | 7.40 | .943 | <.001 |
| Time | Middle occipital gyrus | L | 19 | 1 | -51 | -76 | 5 | 8.11 | .871 | <.001 |
| Time | Middle temporal gyrus | L | 22/42 | 6 | -54 | -40 | 11 | 8.48 | .820 | <.001 |
| Time | Middle temporal gyrus | R | 39 | 1 | 51 | -73 | 17 | 8.99 | .738 | <.001 |
| Time | Superior temporal gyrus | L | 22/42 | 2 | -63 | -40 | 17 | 8.15 | .863 | <.001 |
| Time | WM | R |  | 2 | 9 | -34 | 17 | 10.28 | .523 | <.001 |
| Time | WM | L |  | 3 | -6 | -28 | 20 | 12.54 | .228 | <.001 |
| Time | CSF | R |  | 4 | 12 | -13 | 26 | 10.92 | .419 | <.001 |
| Time | Precentral gyrus | L | 6 | 9 | -36 | -1 | 38 | 14.16 | .113 | <.001 |
| Interaction | Thalamus | L | 18 | 10 | -6 | -49 | -10 | 9.90 | .582 | <.001 |
| Interaction | Thalamus | L |  | 2 | -15 | -34 | 2 | 9.26 | .701 | <.001 |
| Interaction | Thalamus | R |  | 2 | 12 | -28 | 2 | 9.87 | .593 | <.001 |
| Interaction | Thalamus | L |  | 1 | -3 | -7 | 8 | 9.09 | .723 | <.001 |

Abbreviations: BA, Brodmann area; CSF, cerebrospinal fluid; FWE, family-wise error; L, left; R, right; Unc, uncorrected; WM, white matter.

Supplemental Table 12 *Effects of group, time and group×time interaction for right amygdala connectivity during successful inhibition in OCD (n=24) and HC (n=17)*

| Effect | Region | Side | BA | Voxels | X | Y | Z | F | p_FWE_ | p_Unc_ |
| --- | --- | --- | --- | --- | --- | --- | --- | --- | --- | --- |
| Group | Dorsolateral PFC | L | 46 | 2 | -39 | 50 | 11 | 12.21 | .924 | <.001 |
| Group | Anterior cingulate gyrus | R | 24 | 14 | 6 | 23 | 20 | 23.58 | .105 | <.001 |
| Group | Medial PFC | R | 32 | 1 | 3 | 41 | 23 | 13.42 | .840 | <.001 |
| Group | Superior frontal gyrus | R | 10 | 2 | 24 | 47 | 23 | 15.01 | .691 | <.001 |
| Group | Medial frontal gyrus | L | 46 | 8 | -6 | 32 | 38 | 16.00 | .587 | <.001 |
| Group | Precentral gyrus | R | 6 | 2 | 54 | 2 | 47 | 15.34 | .656 | <.001 |
| Group | Pre-SMA | R | 8 | 4 | 3 | 23 | 56 | 17.73 | .424 | <.001 |
| Group | Precentral gyrus | R | 6 | 2 | 36 | -7 | 59 | 14.15 | .775 | <.001 |
| Time | Middle frontal gyrus | R | 46 | 2 | 30 | 29 | 32 | 8.02 | .924 | <.001 |
| Time | WM | L |  | 8 | -21 | -28 | 38 | 14.60 | .099 | <.001 |
| Time | Precentral gyrus | L | 6 | 3 | -27 | -16 | 56 | 9.88 | .640 | <.001 |
| Interaction | Parahippocampal gyrus | R | 30 | 3 | 21 | -34 | -13 | 10.86 | .485 | <.001 |
| Interaction | Superior temporal gyrus | L | 38 | 4 | -51 | -1 | -13 | 13.89 | .141 | <.001 |
| Interaction | WM | R |  | 1 | 36 | -7 | -10 | 7.38 | .967 | <.001 |
| Interaction | Superior frontal gyrus | R | 10 | 22 | 21 | 62 | 8 | 13.56 | .162 | <.001 |
| Interaction | WM | L |  | 4 | -24 | -25 | 38 | 10.41 | .561 | <.001 |

Abbreviations: BA, Brodmann area; FWE, family-wise error; L, left; PFC, prefrontal cortex; R, right; pre-SMA, pre-supplementary motor area; Unc, uncorrected; WM, white matter.

Supplemental Table 13 *Effects of group, time and group×time interaction for left amygdala connectivity during successful inhibition in OCD (n=24) and HC (n=17)*

| Effect | Region | Side | BA | Voxels | X | Y | Z | F | pFWE | pUnc |
| --- | --- | --- | --- | --- | --- | --- | --- | --- | --- | --- |
| Group | Pregenual anterior cingulate | R | 11 | 2 | 9 | 35 | 8 | 12.62 | .911 | <.001 |
| Group | Pregenual anterior cingulate | L | 24 | 8 | -3 | 32 | 11 | 15.16 | .693 | <.001 |
| Group | Operculum | R | 48 | 4 | 39 | -25 | 23 | 14.10 | .794 | <.001 |
| Group | Superior frontal gyrus | R | 46 | 2 | 24 | 44 | 20 | 13.97 | .807 | <.001 |
| Time | Medial frontal gyrus | R | 11/10 | 1 | 15 | 62 | -1 | 8.16 | .958 | <.001 |
| Time | Superior frontal gyrus | R | 10 | 1 | 24 | 62 | 2 | 7.86 | .973 | <.001 |
| Time | Putamen | R | 48 | 7 | 27 | -16 | 11 | 12.12 | .355 | <.001 |
| Time | WM | L |  | 5 | -36 | -52 | 20 | 8.85 | .892 | <.001 |
| Time | Superior frontal gyrus | R | 9 | 40 | 15 | 50 | 35 | 16.62 | .046 | <.001 |
| Time | Middle frontal gyrus | R | 10 | 40 | 27 | 53 | 26 | 11.68 | .412 | <.001 |
| Time | Middle cingulate gyrus | L |  | 13 | -21 | -31 | 41 | 12.04 | .366 | <.001 |
| Time | Middle frontal gyrus | R | 9 | 10 | 30 | 29 | 38 | 10.05 | .703 | <.001 |
| Time | WM | R |  | 4 | 27 | -37 | 38 | 12.94 | .261 | <.001 |
| Time | Middle cingulate gyrus | R | 23 | 2 | 3 | -19 | 41 | 7.68 | .980 | <.001 |
| Time | Precentral gyrus | R | 6 | 4 | 36 | -7 | 41 | 12.31 | .333 | <.001 |
| Interaction | Superior frontal gyrus | R | 10 | 3 | 21 | 65 | 5 | 7.96 | .967 | <.001 |

Abbreviations: BA, Brodmann area; FWE, family-wise error; L, left; R, right; Unc, uncorrected; WM, white matter.

Supplemental Figure 1 *Flowchart for OCD patients and HC throughout the study*


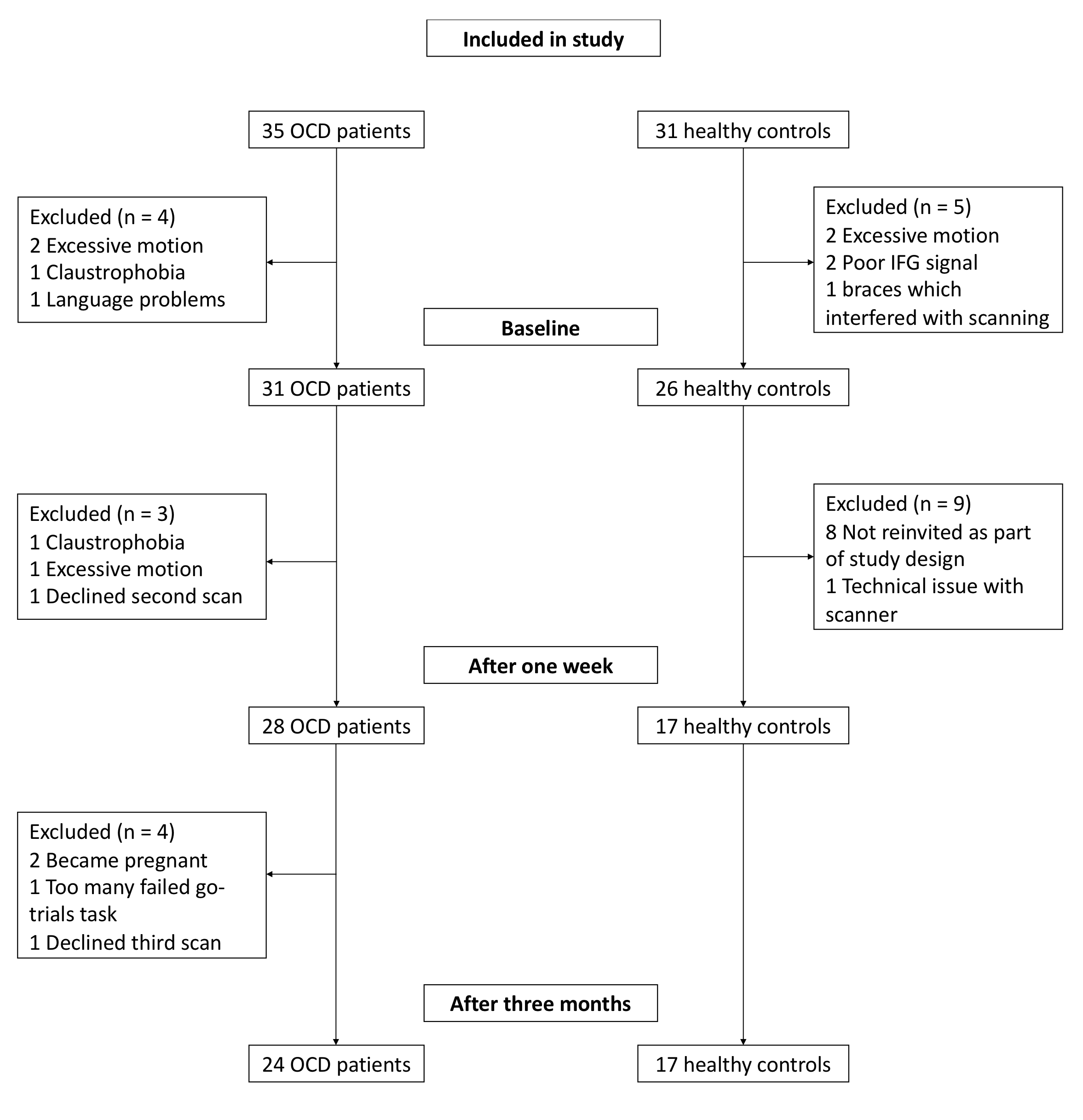


Supplemental Figure 2 *Design of the Stop Signal Task*


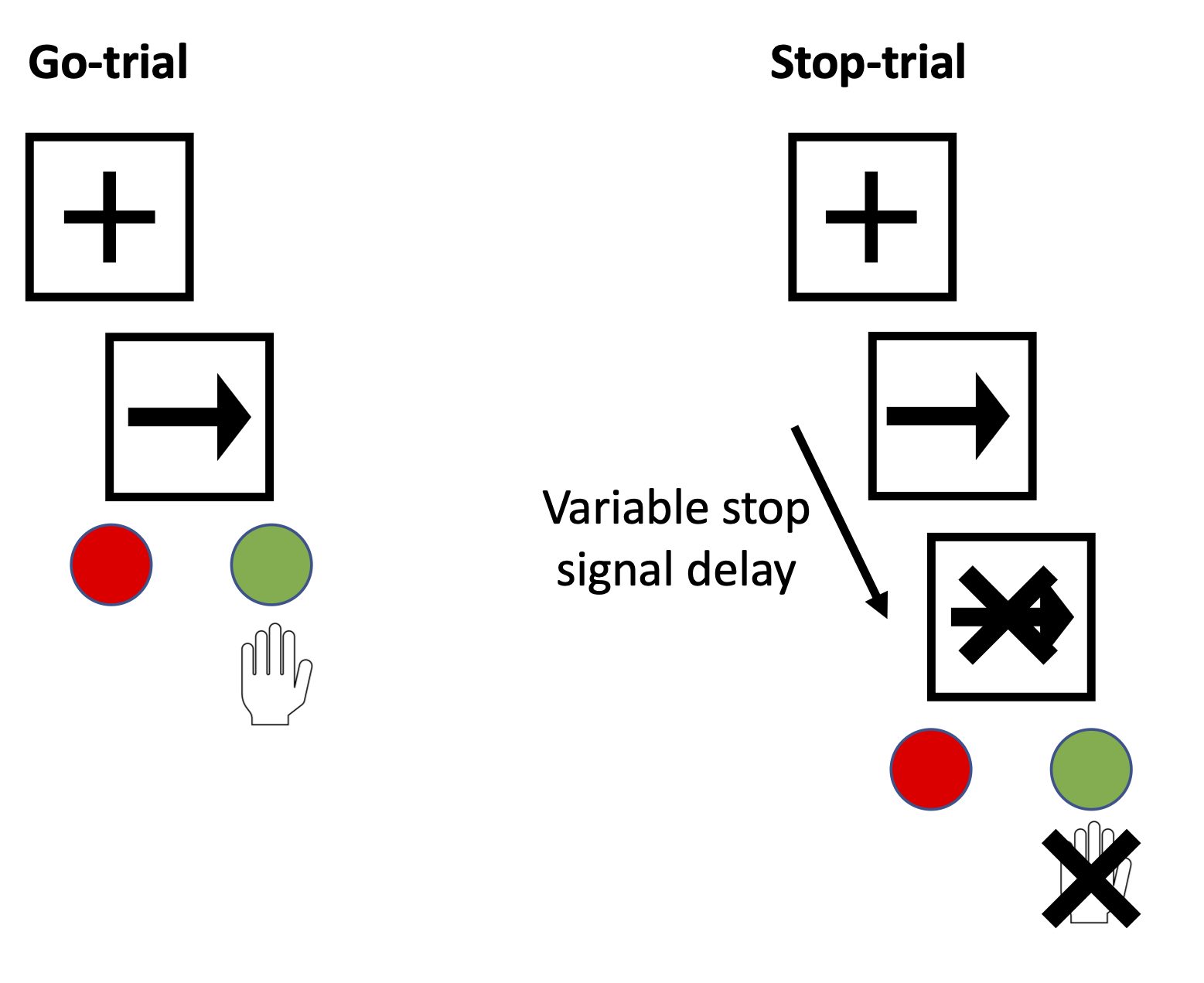


Legend: During go-trials a fixation cross is followed by an arrow indicating which button the participant is supposed to press. During stop-trials a cross is overlaid on the arrow, indicating that the participant is supposed to refrain from responding. The delay between the presentation of the arrow and overlaid cross is systematically varied to ensure that participants are able to successfully respond in about 50% of the trials.

Supplemental Figure 3 *Effect of successful inhibition across all participants at baseline (N=57)*

*
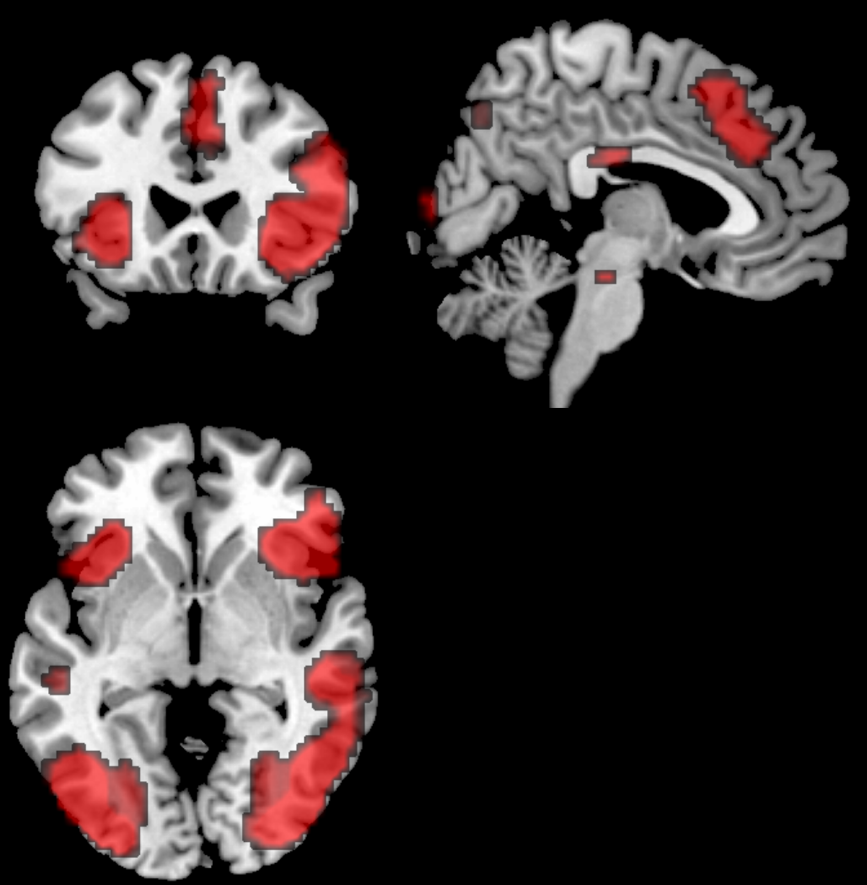
*

Legend: Red voxels indicate significantly more activation during successful inhibition versus successful go-trials.

Supplemental Figure 4 *Effect of successful inhibition at baseline within OCD patients (n=31) and HC(n=29)*

*
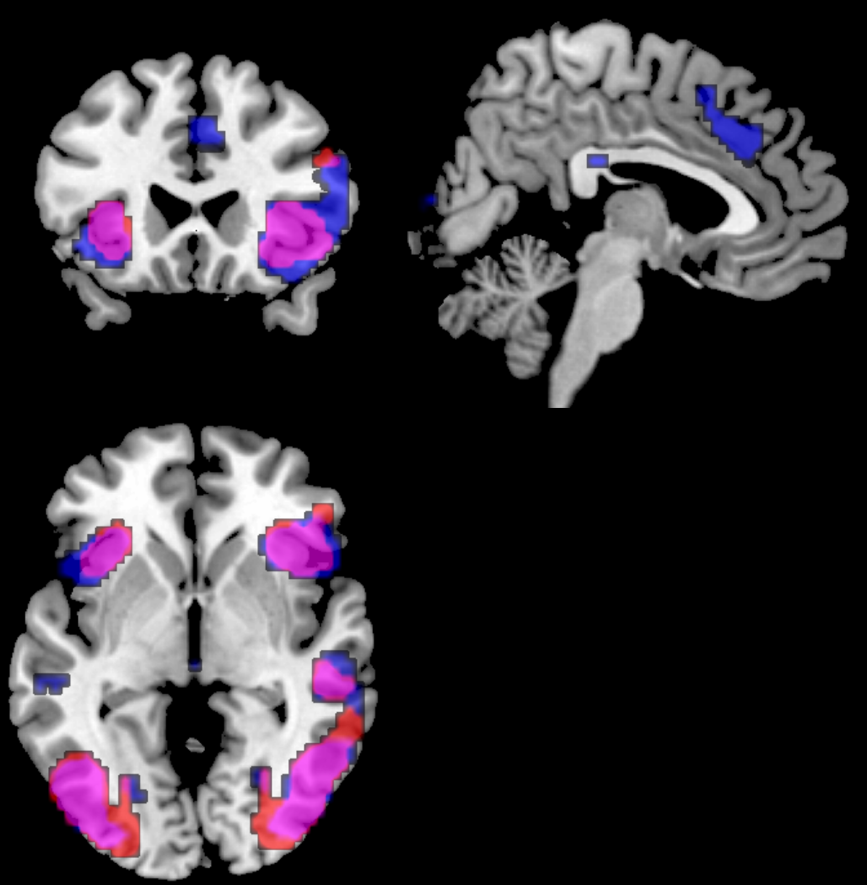
*

Legend: Pink voxels indicate significant activation in both groups, blue clusters indicate significant activation in HC only, and red clusters indicate significant activation in OCD patients only.

Supplemental Figure 5 *Right IFG activation during successful inhibition at baseline in HC (n=26), unmedicated OCD patients (n=24) and medicated OCD patients (n=7)*

Supplemental Figure 5 here

Legend: Figure shows the mean activation in HC, unmedicated OCD patients and medicated OCD patients, along with individual data points. * indicates a significant group difference at p<.05 based on parameter estimates extracted using 6mm spheres, with error bars representing one standard error.

Supplemental Figure 6 *Right amygdala and pre-SMA connectivity during successful inhibition at baseline in HC (n=26), unmedicated OCD patients (n=24) and medicated OCD patients (n=7)*

Supplemental Figure 6 here

Legend: Figure shows the mean connectivity between the right amygdala and pre-SMA in HC, unmedicated OCD patients and medicated OCD patients, along with individual data points. * indicates a significant group difference at p<.05 based on parameter estimates extracted using 6mm spheres, with error bars representing one standard error.

Supplemental Figure 7 *Right IFG activation during successful inhibition at baseline in HC (n=26), adult-onset OCD patients (n=17) and childhood-onset OCD patients (n=14)*

Supplemental Figure 7 here

Legend: Figure shows the mean activation in HC, adult-onset OCD patients, and childhood-onset OCD patients, along with individual data points. * indicates a significant group difference at p<.05 based on parameter estimates extracted using 6mm spheres, with error bars representing one standard error.

Supplemental Figure 8 *Right amygdala and pre-SMA connectivity during successful inhibition at baseline in HC (n=26), adult-onset OCD patients (n=17) and childhood-onset OCD patients (n=14)*

Supplemental Figure 8 here

Legend: Figure shows the mean connectivity between the right amygdala and pre-SMA in in HC, adult-onset OCD patients, and childhood-onset OCD patients, along with individual data points. * indicates a significant group difference at p<.05 based on parameter estimates extracted using 6mm spheres, with error bars representing one standard error
